# Understanding the diagnosis of pre-diabetes in patients aged over 85 in English primary care: a qualitative study

**DOI:** 10.1186/s12875-019-0981-0

**Published:** 2019-06-29

**Authors:** Patrick Burch, Thomas Blakeman, Peter Bower, Caroline Sanders

**Affiliations:** NIHR School for Primary Care Research, Centre for Primary Care and Health Services Research, School of Health Science, Faculty of Biology, Medicine and Health, University of Manchester, Manchester, UK

## Abstract

**Background:**

The benefit of a “diagnosis” of pre-diabetes in very elderly patients is debated. How clinicians manage pre-diabetic blood results in these patients is unknown. This study aims to understand how clinicians are “diagnosing” older patients with pre-diabetic blood parameters.

**Methods:**

Semi-structured interviews and focus groups with health care staff (24 total participants) were conducted in the north of England. Interviews and focus groups were recorded, transcribed and analysed thematically. A grounded theory approach was taken with the theory of candidacy being used as a sensitising concept through which questions were framed and results interpreted.

**Results:**

There is a complex system of competing pressures that influence a clinician in deciding whether, and in what way, to inform a very elderly patient that they have pre-diabetes. The majority of clinicians adjust their management of pre-diabetes to the age and perceived risk/benefit for the patient. Whilst some clinicians choose not to inform certain patients of their blood results, many clinicians maintain, what could be seen as a somewhat paradoxical approach of labeling all older patients with pre-diabetes but downplaying the significance to the patient. The policy, organisational context, workload and professional constraints under which clinicians work, play a significant role in shaping how they deal with pre-diabetic blood results in the very elderly.

**Conclusion:**

There has been recent acknowledgement of how policy and organisational context frames decision-making, but there is a lack of evidence on how this influences uncertainty and dilemmas in decision-making in practice. These findings add further weight for the argument that treatment burden should be included in clinical guidelines.

## Background

Pre-diabetes is a term that encompasses three different biochemical abnormalities: raised HbA1c, impaired fasting glucose (IFG) and impaired glucose tolerance (IGT). Pre-diabetes is common in older patients. The incidence increases with age and in the USA reaches nearly 50% of those aged over 75 [1, 2]. England is currently in the process of a roll-out of a national diabetes prevention programme (DPP). The primary aim of this programme is to prevent people with pre-diabetes from developing diabetes [3]. The programme uses HbA1c or fasting blood glucose results as criteria for entry. The DPP national service specifications state that any patient over 18 with a pre-diabetic blood test result is eligible for the programme. It does not specify an upper age limit for entry.

There is uncertainty around whether some very elderly patients are appropriate to be labelled and treated as pre-diabetic [4]. Pre-diabetes is not a disease state. However, a patient tends not be labelled as having “an HbA1c in the pre-diabetic range”. Rather, the patient “has pre-diabetes”. It is being used as a diagnostic label and, as a consequence, come all the trappings of a fully-fledged disease. This brings with it both positive and negative features. Whilst the labelling of someone with a disease may encourage change and adherence to treatment, it may also cause distress and denial or resistance to treatment [5]. As Yudkin and Montori state when discussing the downsides of labelling patients as pre-diabetic “With this label (of pre-diabetes) comes much of the same baggage as for diabetes, without evidence of long term benefit” [6].

The concept of candidacy has been proposed to describe the multiple patient, clinician and situational factors that combine to decide which patients are deemed suitable for a label such as prediabetes and can access a particular treatment [7, 8]. The concept of candidacy developed from a synthesis of the diverse literature relating to what influences how patients access healthcare, and how patients are deemed “a candidate” for investigations, diagnosis and treatment [9]. The individual factors that, from a clinician’s perspective, come together to create candidacy in this group of patients are examined in this research.

There are guidelines from The National Institute for Health and Care Excellence to help clinicians prevent type 2 diabetes [10]. Whilst they discuss the screening, detection and management of pre-diabetes, they do not address when this may not be appropriate in older patients or those with co-morbidities. American consensus guidelines provide greater guidance and state the following

“Most would agree that a functional and generally healthy 66-year-old individual should be offered diabetes screening since interventions to prevent type 2 diabetes or the complications of type 2 diabetes would likely be beneficial given the presumption of decades of remaining life. Most would also agree that finding prediabetes or early type 2 diabetes in a 95-year-old individual with advanced dementia would be unlikely to provide benefit.” [4]

There is a strong public health argument that we are not picking up pre-diabetes in patients who may benefit from such a label [4, 6, 11]. However, concerns about overdiagnosis and pre-diabetes being used as a diagnostic label have been discussed [12]. Equity of outcome of pre-diabetes treatment has been highlighted as a potential issue for the DPP but there is a considerable opportunity cost to treating patients who may get limited or no benefit [13]. The issues of applying single pre-disease pathways to very elderly patients, who often have co-morbidities, has been highlighted by initiatives suggest as the Choosing Wisely campaign and the Realistic Medicine movement in Scotland [14–16]. These initiatives point out that practice, that may be appropriate and evidence based in younger patients, may cause harm and unnecessary treatment burden in older patients.

The focus of this paper is to understand how clinicians label and discuss HbA1c results with patients with pre-diabetes at the extreme of old age. There is no widely accepted definition of very old but several studies have used 80–85 as a cut off for the extreme of old age [17, 18], our questioning therefore focused on patients above 85.

## Methods

### Design

This research was carried out as part of a broader piece of research into how primary care clinicians treat patients with pre-diabetes and how and why they refer patients to the NHS DPP. Ethical approval was given by the North West Greater Manchester East ethics committee (17/NW/0426). A combination of one-to-one semi-structured interviews and focus groups were used [19]. This combination has been used in other health services research and enables insights that come from practitioners interacting and discussing complex areas of practice, whilst also allowing pragmatically for flexibility and some more in-depth insights from individual interviews [20–22]. We felt that focus groups provided an environment that encouraged clinicians to share usual practice and admit to, and discuss, negative feelings that may not have been shared during an individual interview [23, 24].

**Fig. 1:**
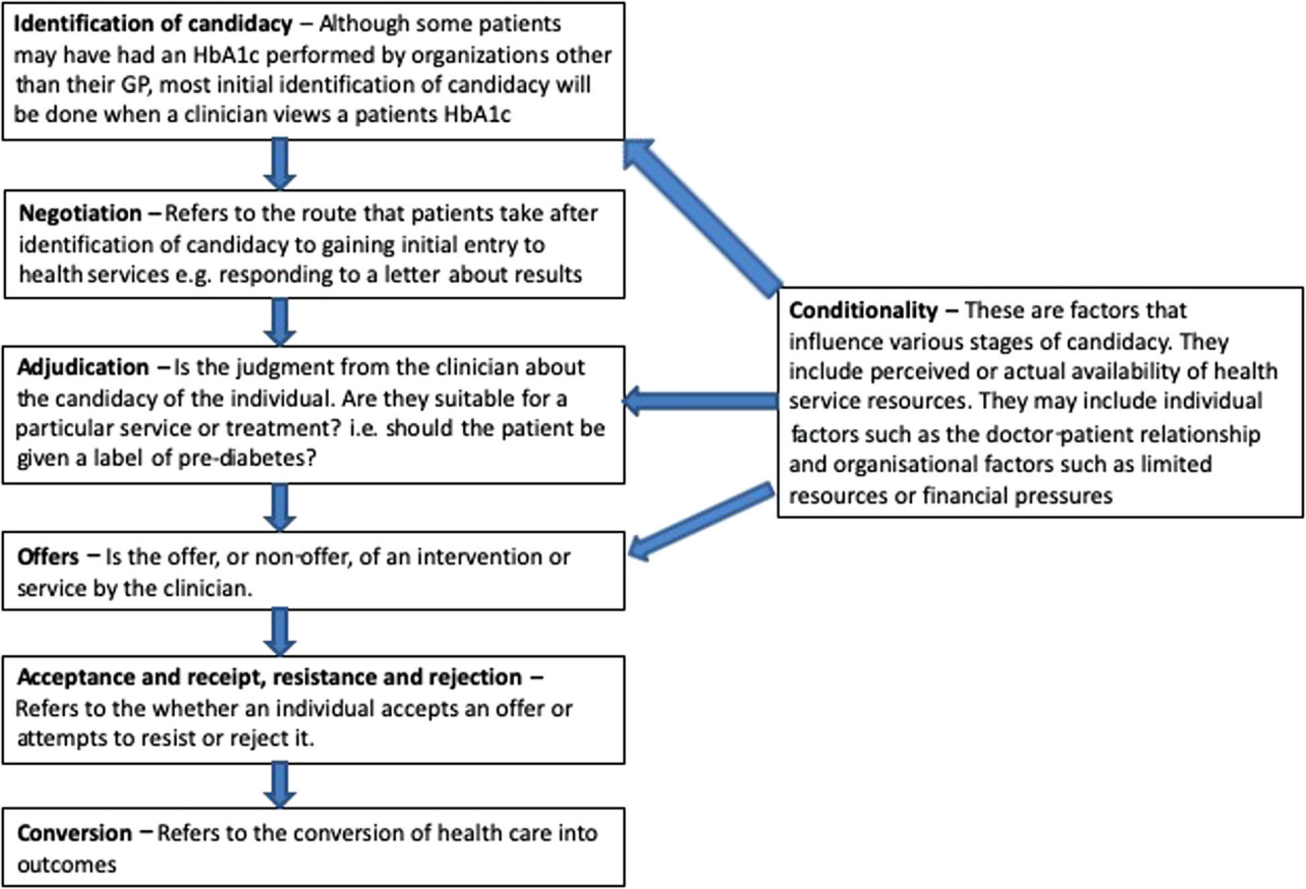
Key aspects of candidacy and how they relate to the diagnosis of pre-diabetes

Wider research by the DPP evaluation team focused on the patient, as well as clinician, perspective of accessing the DPP. Candidacy was used as a sensitising concept to guide the wider research project and highlight particular issues with access for certain vulnerable groups. Whilst the theory is generally used for exploring underutilisation of healthcare in disadvantaged groups, the concepts that make up the theory are equally useful in thinking about any type of healthcare usage in any group of patients. We have used the lens of candidacy to view the clinician’s role in diagnosis, labelling and treatment of pre-diabetes in extreme age. Figure 1 shows key aspects of the theory and how it relates to diagnosis of pre-diabetes.

### Participants and recruitment

Seventeen semi-structured interviews and two focus groups were carried out with healthcare staff between November 2017 and July 2018. None of the group participants were interviewed on a one-to-one basis. A total of 24 participants from 15 different practices in the midlands, north-west and north of England took part. Purposive sampling was used to select participants with a wide variety of personal characteristics. We tried to have maximum diversity in the length of primary care experience of the participants, sex of participants, the size and location of practices and the socio-economic background of practice populations. As health promotion and pre-diabetes is not dealt with exclusively by GPs, we recruited a mixture of primary care professionals to interview. Apart from one health ambassador/advocate, all participants were clinically trained. The characteristics of participants and the practices they work in are listed in Tables 1 and 2. Some participants were recruited from personal contacts of the wider research team; others were recruited via “cold” telephone or email contact to practices.

**Table 1:** Characteristics of study participants

Professional status	
GP	11
GP Registrar(trainee)	1
Nurse practitioner	2
Practice nurse	8
Health care assistant	1
Patient advocate	1
Sex	
Female	14
Male	10
Primary care experience	
< 5 years	7
5–15 years	7
> 15 years	10

**Table 2:** Characteristics of the principal practice in which participants worked

Practice size (number of patients)	
< 5000	5
5000–10,000	10
10,000-15,000	8
15,000+	1
Practice location	
Rural village	2
Rural town	5
Urban city and town	9
Urban major conurbation	8
Socioeconomic deprivation status of practice	
In 20% of least deprived practices	7
Between the top and bottom 20%	6
In 20% of most deprived practices	11

### Data collection and analysis

Interviews were based on a topic guide drawn up prior to commencement that included questions relating to different aspects of primary care management of pre-diabetes. Table 3 shows the part of the topic guide relevant to this paper.

**Table 3:** Topic guide

Health practitioner role and diabetes prevention at the practice	
What is your role at the practice and what do you do in relation to diabetes prevention and management?	
Who provides diabetes prevention mostly in this practice?	
How important is diabetes prevention in primary care?	
How do you/your practice deal with a new pre-diabetic blood result?	
Discussing and dealing with heightened risk of type 2 diabetes in consultations	
Do you think the language you use to describe risk is important?	
Do you think there are any barriers to providing diabetes prevention advice during a consultation?	
Do you tell all patients if they have pre-diabetes? Are there certain groups, such patients aged over 85, in which you would not disclose a pre-diabetic HbA1c?	
Do you approach risk explanation the same in all patients eg. education/age?	
What type of information do you provide when first discussing heightened risk?	

All focus groups and most one-to-one interviews were conducted face-to-face. Two of the one-to-one interviews were conducted via video call and one via phone. Interviews lasted between 19 and 60 min. The interviewer (PBu) is a practicing GP and academic. The focus groups (size of each group: 4 participants and 1 facilitator) were taken from individual practices and consisted of a mixture of GPs and nurses who worked together in the same practice. The groups were facilitated by PBu. Audio recordings of the interviews and focus groups were transcribed by a professional transcription service using intelligible transcription. Nvivo 11 software was used to collate, analyse and code data.

Thematic analysis of the data was conducted using a constant comparison approach [25, 26]. Themes were identified in interviews that were then further explored with subsequent subjects. As more data emerged, themes/categories of data were refined. A point of data saturation was reached before the commencement of the focus groups. Initial data analysis and coding was carried out by PBu. CS and TB commented on original transcripts and coding. The codes and overarching themes were refined through discussion between PBu, CS and TB.

## Results

We present below how pre-diabetes is managed by different clinicians and practices. We then discuss the main themes that arose when discussing whether to inform, and/or how to inform and treat, a very old patient with pre-diabetes.

**Fig. 2:**
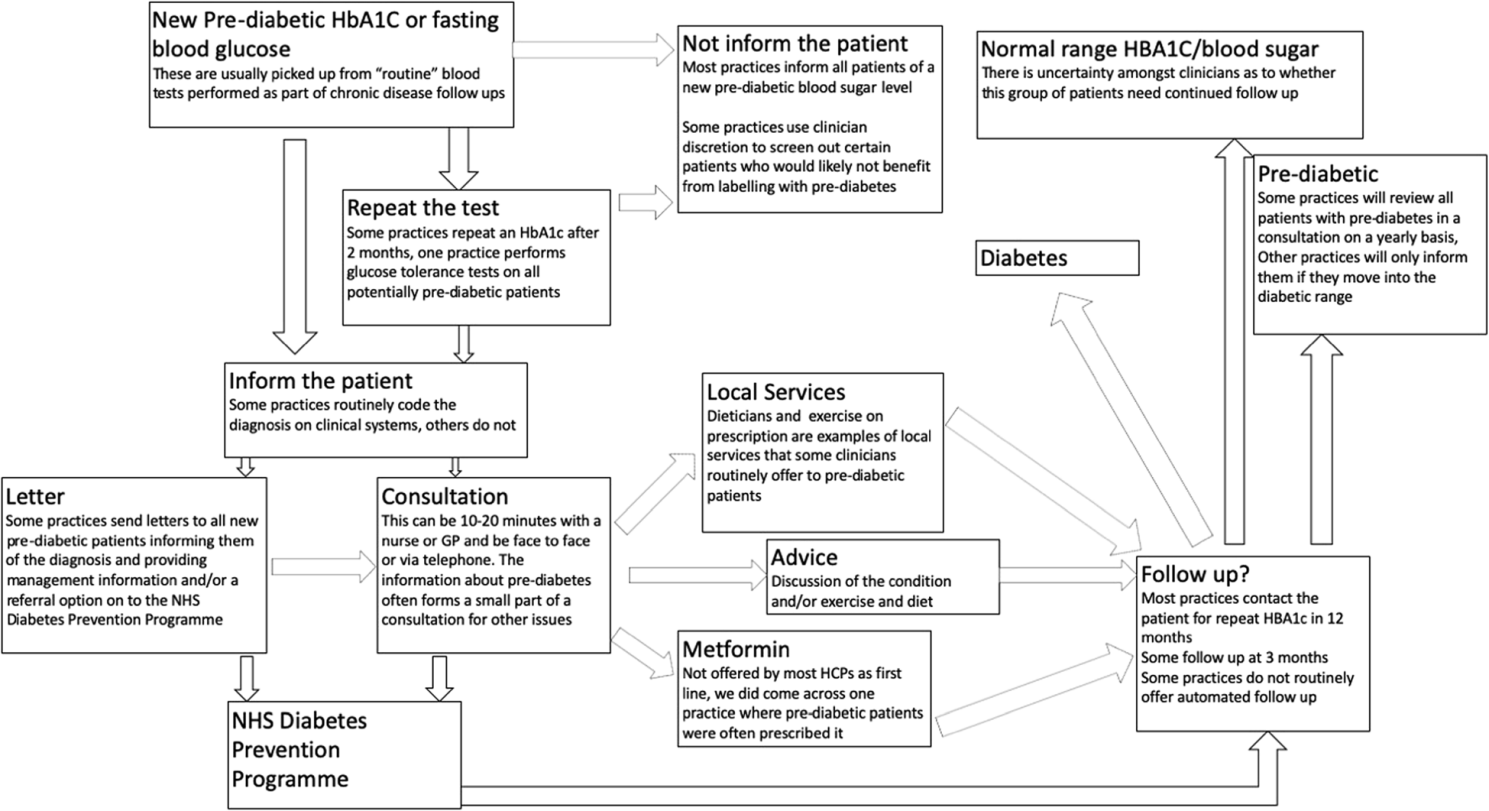
Pathways in the diagnosis and management of pre-diabetes

Figure 2 uses the information given to us by the study participants to illustrate the variety of ways in which pre-diabetes is managed in different practices and by different clinicians. The start of a patient’s pre-diabetic “journey” is when they undergo an HbA1c or FBG test. Apart from one nurse practitioner, the non-GP respondents were not involved in filing blood tests and had no influence on which patients would have been labelled as pre-diabetic by the clinician who had filed their blood results. Although some HbA1c testing is opportunistic and based on a clinician’s assessment of risk, our participants reported that most of it happens as part of routine disease monitoring. All interviewed participants had a role which involved informing patients that they had pre-diabetes. For most of the nurses, the HCA and the patient advocate, the adjudication that a patient should be labelled as pre-diabetic was not made by them. However, most had some degree of autonomy as to how that diagnosis was relayed to the patient and what treatment or recommendations the patient was given.

### “It depends on the patient” - identification, adjudication and offers of treatment using a person-centred approach

There was variation in whether clinicians informed the patient of the diagnosis, how they informed the patient of the diagnosis and whether they offered treatment/advice or referral on to the DPP to very old patients. Concerning the disclosure of a pre-diabetes diagnosis to very elderly patients, some professionals would not always inform. Nevertheless, there was a split in our participants. Seven of our GPs reported that they will sometimes not inform very elderly patients of pre-diabetic blood results. The adjudication, of whether to inform a patient of their results, generally took place when filing blood results but sometimes during consultations. Most other GPs and nurses in our sample appear to recognise that certain patients may not benefit from being told they have pre-diabetes but, nonetheless, they still support informing them.

*I: a 92 year old woman with co-morbidities and she’s got an HbA1c of 43, would you tell her?*

*R: Yes. Well, unfortunately, they’ll get a letter out from the surgery saying to make an appointment with the nurse. So, they would still get told, although it’s not my opinion that they always should be told, for the purpose of a referral [to the DPP]. But, I think it’s good that people should all be aware, because I’d like to know at any age. Practice nurse 3*

Many clinicians maintain an approach of informing all older patients but then downplaying the significance of the result. Non-GP respondents appeared more likely to “follow the protocol” and give a full explanation of pre-diabetes, its implications, and its treatment, to all patients, regardless of age.

*I: do you tell everyone that they’ve got pre-diabetes, or are there certain patients that you would not?*

*R: I don’t think I haven’t really. The obvious ones would be the very elderly or the people with pretty severe dementia. But even then, there is again, somebody who’s very ill, has somebody looking after them, so we would normally pass that information on. … So, you would say, well we picked this condition up, this is what it means, and you could perhaps have a look at the food that they’re eating…but equally being realistic as to what that means.* GP 1

Most of the clinicians were uncertain of the benefits of “diagnosing” and treating pre-diabetes in older patients. In the main, it was felt that treating the very elderly for pre-diabetes was likely to be of limited use to the patient. Clinicians expressed having varying amounts of knowledge of pre-diabetes and of the likelihood of progression to diabetes, particularly the risks in the very elderly. There was no consistent criteria that clinicians used to grant (or not grant) candidacy to an elderly patient but factors such as co-morbidities, functional ability and life expectancy were frequently mentioned. This uncertainty about the evidence base, and the benefits of a diagnosis, created a dilemma in whether or how to communicate the diagnosis with patients.

*“I think it depends on the patient, but I think the reward for telling somebody who’s over 85, say, I think are going to be lower and lower …I may play it down much more, I think, in more frail elderly patients”* GP 4

In the main, clinicians worked towards minimising the impact of the diagnosis in the very elderly. They adjusted the type and intensity of advice and may have decided whether to refer on to the DPP depending on their perceived benefit to the patient. This approach appears to “tick the box” of diagnosis and having “done the job” whilst trying to impact the patient as little as possible. The patients may have a diagnosis of pre-diabetes but they were seen to be unlikely to convert this diagnosis into improved health outcomes. With younger, fitter patients several clinicians state they would be more likely to talk about the risk of progression to diabetes and discuss lifestyle changes in more detail.

The language used to describe pre-diabetes varied amongst clinicians. Some actively “diagnosed” patients with pre-diabetes whilst others avoided the phrase and tended to use phrases such as “your sugars are a bit on the high side” or “borderline diabetes”. Some varied their phraseology depending on their perceived benefit of diagnosis to the patient. Many were less inclined to discuss the progression to diabetes and potential complications in very elderly patients and where they offered referral to the DPP, were less likely to “sell” it than they would to a patient with greater perceived benefit.

*“So I’ve got somebody, you know, somebody, if they’re much later in life, I’d say, you know, to some extent I would say, you’ve got this far, it’s just a matter of keeping the sugar intake down. Again, not wanting this to get, sort of, progress on more.” GP 5*

The desire to avoid unnecessary anxiety or harm was mentioned by most clinicians as a reason for not informing some older patients or for “down playing” the impact of pre-diabetes.

*“So I suppose I think patients do have a right to know about their health when it’s going to affect their health, but if you weigh it all up as a doctor and you think actually, this is going to cause more harm than help, because this is a 95 year old that’s really anxious and already struggling with some other medical problems … then you’ve got to think really, why would you tell that person?*” *GP 11*

### “As long as you tick the box” - policy and organisational context in the diagnosis of pre-diabetes

As set out in the introduction and in Fig. 2 the pathway to DPP referral has been constructed as a direct response to policy pressures to address rising rates of type 2 diabetes; although there are variations in pathways to referral via individual practices and practitioners. Some practices send out letters offering a place on the DPP to electronically identified patients with pre-diabetes. In some practices, CCG personnel have come into practices to run searches for patients with pre-diabetic HbA1Cs. These patients have then been sent information offering them referral onto the DPP or called into the practice to discuss their results. In some cases, these patients have not been previously informed that they had abnormal blood sugar levels. The findings from analysis of qualitative data revealed how the organisational approach to diagnosis and treatment can make it hard to treat patients as individuals that will differ in the benefit and harm they receive from a diagnosis of pre-diabetes. The exchange below from a focus group highlights the problems of individual clinicians taking a decision not to inform a patient of their pre-diabetic blood result when a practice system is in place that will automatically tell the patient.

*“GP8: I have had a couple recently where I’ve thought, shall I tell them that their HbA1c is 44? No, let’s address that at a different time.”*

*GP9: Is that why I picked them up that say, nobody ever told me?*

*“GP8: Probably, yeah, when they get this letter out from the recall.”* *Focus group 1*

In some of the areas, practices were being financially incentivised to diagnose patients with pre-diabetes and/or refer onto the DPP. In two of the sampled practices, automated systems were put into place as a direct response to the financial incentivisation of pre-diabetes case finding. The comment below came from a GP in an area where a local scheme exists that pays practices for each patient they diagnose as pre-diabetic who has a documented BMI and receives lifestyle change advice.

*“[when discussing why the clinicians in the group would still identify and assess a 95-year-old patient with dementia in a nursing home] Yeah, rather than what the patient’s age is, we’ve got to show that we are identifying these results, we are providing the health education and doing the relevant health checks for these patients.”* GP in focus group 2

### “A lot of other issues” -professional obligations and workload conditions

The issue of a new pre-diabetes diagnosis often comes up during review appointments for other problems, rather than during a separate appointment to address pre-diabetes. Several GPs and practice nurses felt the context in which they had to inform the patient of the result influenced whether or not and in what way they informed the patient. They were less inclined to inform and discuss the implications of pre-diabetes fully when they had other clinical priorities.

*“I: an 89 year old lady that comes with a HbA1c of 43 would you refer her to the programme?”*

*“R1: I don’t think I would. I don’t…I don’t know, because normally that might be thrown in with a lot of other things that’s in that consultation I’d have thought. I’m just thinking of maybe the sort of ones that you pick up, you might be running a HbA1c and other bloods because of an acute presentation and actually that result isn’t something that really worries you.”* GP in focus group 1

One locum GP reported that she was more likely to disclose a pre-diabetic blood result to older patients because of time pressures.

*“I think if you’re very busy on a certain day, you might just see the result, you might say, right, that’s pre-diabetic, just check they don’t already know that, right, we better just call them in, you know, that, kind of, just on auto pilot, but I think on another day where you have more time, you might look more at that patient, so if you knew that patient better, if you knew what other kind of problems they had, if you knew how old they were, all those factors might play a role in whether you let them know or not.”* GP 11

With care often provided by multiple professionals and automated systems, the fear amongst many clinicians is that not informing patients may generate problems in the future. Fear of complaint and medico-legal consequences were raised by several clinicians.

*“…I don’t even think we’re in any position to think about, oh, is it even worth the benefit for this, because unfortunately people complain about lots of things and they can even see their own records and question it and all that sort of thing. So it’s just as easy just to be like, well, we’ve told you.”* GP 7

## Discussion

### Summary

There is a network of competing pressures that influence a clinician in deciding whether, and in what way, to inform a very elderly patient that they have a pre-diabetic blood test result. The majority of clinicians tended to adjust their management of pre-diabetes to the age and perceived risk/benefit to the patient. Whilst some clinicians choose not to inform certain patients of their blood results, others maintain, what could be seen, as a somewhat paradoxical approach of informing all older patients that they have pre-diabetes but downplaying the significance of the result where they feel it does not benefit the patient. Whilst the “diagnosis” may be recorded on clinical systems, a referral to the DPP would not usually be generated and it is unlikely this approach converts to any beneficial health outcome for the patient.

When viewed through the lens of candidacy, it is apparent how important “conditionality” is in influencing an individual clinician to grant, or not to grant, candidacy to an elderly patient. Whilst a clinician needs to adjudicate and make an individual judgement as to how suitable a patient is for a diagnosis or treatment, there are many external factors influencing that decision. A personalised approach to pre-diabetes management can be undermined by the policy and organisational context in which the clinician works in and the workload and professional constraints under which they practice. Such pressures appear to make clinicians more inclined to disclose and, at least partially, discuss a pre-diabetic blood test result, even when they feel it may not be in the patient’s best interest.

Whilst we are not advocating that clinicians “hide” abnormal results from patients, it needs to be recognised that non-disclosure of abnormal test results to patients takes place [27]. As may be the case with a stable mildly lowered eGFR in a patient aged over 85, this is often done to reduce information/treatment burden when there is judged to be no benefit to the patient [28]. These acts of clinical paternalism undermine patient autonomy to try and maximise benefit (as judged by the clinician) in an often time pressured environment [29]. A full discussion of the ethics behind this approach to patient care is beyond the scope of this paper. When clinicians do inform patients that they have pre-diabetes, they need to be fully informed so that a shared decision-making process can take place. Adopting a half-way house of disclosing the condition whilst playing-down the significance and attached risks may not have the desired effect of reassurance. It also takes time and could still add to treatment burden for some patients.

### Strengths and limitations

The sample of clinicians had a variety of roles in primary care and came from a wide variety of locations in England. We recognise that the accounts were actively constructed though interaction between participants and a GP Researcher (PBu). Although some respondents may have felt freer to talk to a fellow clinician, as has been seen in other qualitative work, PBu was sometimes seen as an “expert” or “judge” by some of the respondents [30] Further exploration of the topic will benefit from patient participant accounts.

### Comparison with existing literature

To date, there is little research exploring how diagnosis and disclosure of pre-conditions are managed in general practice. The work that exists has focused on patient and professional perspectives in the non-disclosure of Chronic Kidney Disease (CKD) [27, 28]. The 2012 qualitative work by Blakeman et al. examined the perspectives of clinicians disclosing early stage CKD to older patients. Their findings echoed many of the findings in this study in terms of concerns of inducing unnecessary anxiety and the use of reassurance when disclosure occurs.

A USA research study examining the attitudes of primary care clinicians to pre-diabetes has shown that views vary significantly [31]. Some, but not all, clinicians believe the usefulness of diagnosis depends on the patient’s individual context, with some being dissuaded from diagnosis in those with co-morbitidites and other cardiovascular risk factors [32]. Existing guidelines and pathways in the UK ignore patient age and co-morbidities and see diagnosis and disclosure of pre-diabetes to patients as a simple function that will occur on the wider pathway to self-management [10].These guidelines do not take account of treatment burden. Patients may feel a moral imperative to self-manage disclosed “conditions” but struggle to do so and get little to no benefit if they do [33, 34]. The use of reassurance and down-playing of the condition may not always be interpreted by the patient in the way in which the clinician intended. Studies of reassurance given by clinicians have found that when patient perspective is not acknowledged then reassurance is often unsuccessful [35].

Recent work has looked at the literature around potential causes of overdiagnosis and categorised the drivers under the headings of cultural, health system, industry and technology, professional and patient/public [36]. The themes found in our work mirror many of the drivers described in the existing literature. Our approach of using candidacy as a lens to view the diagnostic and therapeutic process, where the risk for overdiagnosis exists, adds to this work. Whilst there has been acknowledgement of treatment burden and need to recognise this in guidelines, there has been a lack of insight into perspectives based on everyday decision-making in practice [34].

Candidacy is a theory that is most often applied to understand the lack of access and use of healthcare for certain vulnerable groups [7, 9]. We have demonstrated that candidacy can be a useful framework through which to examine the drivers of overdiagnosis, in the context of pre-diabetes in the very elderly. Our work highlights the pressures that can influence an individual clinicians’ adjudication of candidacy. It highlights issues in the screening for and identification of pre-diabetes in the very elderly. It also highlights how far person-centered care can drift when only the clinician/clinical system have the power to grant or deny access to care. We have shown that policy and organisational pressures can create candidacy in patients who will not receive improved health outcomes as a result. Use of this theory with modifications may aid further studies looking at what factors drive overdiagnosis and the effects of the tensions that can be produced by using a standardised, population-based approach in primary care.

## Conclusions

The risk of overdiagnosis should not take away from the risk of missing opportunities to identify pre-diabetes in those that may benefit from intervention and lifestyle change. However, there is limited benefit and potential harm in diagnosing and treating some very elderly patients, and the numbers of these patients who would fulfil the criteria for pre-diabetes is probably large. The opportunity cost of treating these patients in an overstretched primary care service is not insignificant.

A more nuanced approach needs to be taken that maximises the utility of HbA1c testing and minimises the potential for treatment burden and unnecessary clinical workload. Policies and practice that leads to routine HbA1c testing in asymptomatic patients in the final years of their lives, who would not benefit from a diagnosis of type 2 diabetes, needs to be evaluated. Avoiding unnecessary HbA1c testing, where appropriate, may reduce tensions and decision making in subsequent patient encounters. In depth understanding of routine clinical practice through a systems-based learning approach needs to inform current guidelines [37]. Participatory methodologies for development of guidelines such as the RAND/UCLA Appropriateness Method may help navigate the challenge of overdiagnosis and through a rigorous consensus process lead to guidance that fits more readily with routine clinical practice [38–40]. Policies and practice at all levels should be reviewed in order to legitimise, rather than undermine, clinicians in taking account of treatment burden within the adjudication process.

## Data Availability

The data generated and analysed during the current study are not publicly available due issues of confidentiality but are available in annoymised form from the corresponding author on reasonable request.

## Abbreviations

BMI: Body mass index
CCG: Clinical commissioning group. A body responsible for the planning and commissioning of health care in a local area of England
CKD: Chronic kidney disease
DPP: Diabetes prevention programme
eGFR: Estimated glomerular filtration rate
GP: General practitioner (family doctor)
HbA1c: Glycated haemoglobin, a marker of your average blood sugar level of the preceeding two to three months
IFG: Impaired fasting glucose
IGT: Impaired glucose tolerance
RAND: Research and Development Corporation, an American non-profit policy think tank
UCLA: University of California, Los Angeles
